# Association of vitamin A deficiency with early childhood stunting in Uganda: A population-based cross-sectional study

**DOI:** 10.1371/journal.pone.0233615

**Published:** 2020-05-29

**Authors:** Paddy Ssentongo, Djibril M. Ba, Anna E. Ssentongo, Claudio Fronterre, Andrew Whalen, Yanxu Yang, Jessica E. Ericson, Vernon M. Chinchilli

**Affiliations:** 1 Center for Neural Engineering, The Pennsylvania State University, University Park, State College, PA, United States of America; 2 Department of Engineering Science and Mechanics, The Pennsylvania State University, University Park, State College, PA, United States of America; 3 Department of Public Health Sciences, The Pennsylvania State University College of Medicine, Hershey, PA, United States of America; 4 Center for Applied Studies in Health Economics, The Pennsylvania State University College of Medicine, Hershey, PA, United States of America; 5 Centre for Health Informatics, Computing and Statistics, Lancaster University, Lancaster, United Kingdom; 6 Department of Pediatrics, The Pennsylvania State University College of Medicine, Hershey, PA, United States of America; Addis Ababa University School of Public Health, ETHIOPIA

## Abstract

**Background:**

Despite the high prevalence of childhood protein-energy malnutrition and vitamin A deficiency in sub-Saharan Africa, their association has not been explored in this region. A better understanding of the epidemiologic link could help define effective preventive strategies. We aimed to explore the association of vitamin A deficiency (VAD) with stunting, wasting, and underweight among preschool children in Uganda.

**Method:**

We analyzed a population-based, cross-sectional data of 4,765 children aged 6–59 months who participated in 2016 Demographic and Health Surveys conducted in Uganda. We utilized generalized linear mixed-effects models with logit link function, adjusting for potential confounders to estimate associations between VAD and stunting, wasting, and underweight.

**Results:**

The prevalence of VAD was 8.9% (95% CI: 8.1% to 9.6%, n = 424). Twenty-seven percent were stunted (95% CI: 26.1% to 28.6, n = 1302), 4% wasted (95% CI: 3.6% to 4.7%, n = 196), and 17% underweight (95% CI: 16.0% to 18.2%, n = 813). After adjusting for household factors (e.g., wealth index, education and working status of parents, owning land for agriculture, livestock, herds, or farm animals), vitamin A supplementation, and community factors (e.g., population density, crop growing season lengths, place of residence), children with VAD had 43% higher odds of stunted growth than those without VAD (adjusted odds ratio, 1.43 (95% CI: 1.08 to 1.89, p = 0.01). No association was observed between VAD and wasting or underweight.

**Conclusion:**

Vitamin A deficiency was associated with higher odds of stunting, and the association was independent of the individual, household, and community-level variables.

## Background

Globally, vitamin A deficiency (serum retinol < 0.70 μmol/L) (VAD) is estimated to affect over 127 million preschool-aged children [1], more than one-third in sub-Saharan Africa [2]. This age group is particularly susceptible due to the lack of dietary diversity and a high burden of diarrheal and helminthic diseases. In sub-Saharan Africa, dark green leafy vegetables, yellow/orange sweet potatoes, carrots, and orange and yellow fruits are the main sources of vitamin A, particularly among households from lower socioeconomic status who cannot afford animal sources of vitamin A such as eggs and meat. However, the bioavailability of provitamin A carotenoids in dark green leafy vegetables is not high enough to meet the recommended dietary allowance of vitamin A for children aged 1–3 y, of 300 μg retinol activity equivalents (RAE) per day [3, 4]. VAD disorders such as diarrheal diseases, respiratory diseases, and immune disorders lead to approximately 2.5 million preventable deaths annually [5, 6] with most of these deaths occurring in sub-Saharan Africa [1]. High dose supplementation with vitamin A (200, 000 IU for children aged 12–59 mo, 100 000 IU for children aged 6–11 mo) 1–3 times per year reduces the risk of all-cause child mortality by an average of 24% [7]. The decrease in mortality is observed in deaths attributable to diarrheal diseases and measles.

Stunting (length/height-for-age Z score [HAZ] <-2 based on the World Health Organization (WHO) Child Growth Standards), wasting (weight-for-length/height Z score [WHZ] <-2) and underweight (weight-for-age Z score [WAZ] <-2) are responsible for over 45% of under-5 mortality and impaired cognitive development [8] and are associated with multiple risk factors, including fetal growth restriction [9], enteric and systemic infections [10], diarrheal diseases [11] and poverty. Given that these multifactorial risk factors are highly concurrent in individuals with VAD [12], it is plausible that VAD plays a critical role in the occurrence of undernutrition. Findings from experimental studies suggest that vitamin A may affect growth through the regulation of growth hormone (GH) and thyroid-stimulating hormone beta genes. Deficiency of retinoic acid is associated with reduced secretion of GH from the pituitary gland, [13, 14] and causes a marked reduction in body weight in rats. [15]. Historically, the nutritional status of children was assessed by a classification based on the deficit in weight-for-age, initially proposed by Gomez in 1956 and a decade later modified by Jolliffe [16, 17]. However, in November of 1970, the Joint FAO/WHO Expert Committee on Nutrition emphasized the need for distinguishing between acute and chronic under-nutrition. A few years later, McLaren & Read [18] and Waterlow proposed [19] a growth failure classification based on weight and height and age and weight-for-height, respectively. Subsequently, an FAO/UNICEF/WHO Expert Committee recommended the use of height-for-age and weight-for-height as primary indicators of the nutritional status of children [20, 21]. These anthropometric indices of child growth, reflect distinct biological processes; length/height-for–age reflecting linear growth but weight-for-age or weight-for-height/length reflecting ponderal growth.

To date, few studies have explored the association of VAD with stunting, wasting, and underweight. These studies often have yielded inconsistent findings. Among preschool children in Indonesia, fortification of monosodium glutamate with vitamin A improved height but not the weight of children under 5 y [22]. Similarly, in another study, supplementation with a 214 μmol vitamin A (206000 IU/L, or 107 μmol vitamin A/L if aged <12 mo) improved the linear growth of children but not ponderal growth in Indonesia [23]. On the contrary, vitamin A supplementation did not affect growth in clinical trials in China [24] and India [25]. These findings suggest that studies delineating the association of VAD and growth failure ought to be population-specific, and finding from one population may not be generalizable to another. A meta-analysis that evaluated the effect of vitamin A or carotenoid supplementation during pregnancy on maternal, fetal, neonatal and early infant health outcomes failed to include child undernutrition in the analysis due to the lack of sufficient studies assess the association of VAD and undernutrition [26]. Therefore, in the context of the high prevalence of VAD and mortality from childhood undernutrition in sub-Saharan Africa, addressing the potential association between VAD and stunting, wasting, and underweight is timely. Using a nationally representative cross-sectional data of Uganda, we investigated the association of VAD with stunting, wasting and underweight, and hypothesize that VAD is associated with growth failure in children in Uganda.

## Methods

### Data sources and participants

Data were from the latest (2016) Uganda Demographic and Health Survey (UDHS). The data were collected between June 20, 2015, and December 16, 2016, by the Uganda Bureau of Statistics in collaboration with the Ministry of Health and coordinated by ICF international in Rockville, Maryland, USA [27]. International Development (USAID), Government of Uganda, the United Nations Children’s Fund (UNICEF), and the United Nations Population Fund (UNFPA) provided financial support to carry out the DHS program. The survey collected nationally representative health data to monitor and evaluate population health and nutrition programs. The details of sampling methods are discussed in detail elsewhere. [27] In summary, data collection involved a multistage stratified sampling design. First, Uganda was divided into 15 regions. Within these regions, populations were stratified by urban and rural areas of residence. Within these stratified areas, a random selection of 696 enumeration areas (EAs) or primary sampling units (PSUs) were drawn. In Uganda, an EA is a geographic area that covers an average of 130 households. PSUs were selected based on a probability that was proportional to the population size. Next, in the second stage of sampling, all households within a PSU were listed from the most recent population census (2014), and ~30 households per PSU were randomly selected for an interview with the use of equal probability systematic sampling. In total, a representative sample of 20,880 households were selected for the 2016 UDHS. For each sampled household, household members were listed, and women who were eligible for a more-detailed interview were identified. These women were between the ages of 15 and 49 years. Height and weight information were also collected from eligible women and men, as well as children of ages 0–59 months. Blood samples were collected from children of ages 6–59 months for laboratory testing of blood retinol-binding protein, a surrogate marker for vitamin A concentration.

### Assessment of undernutrition status (outcome)

We analyzed the data for stunting, wasting, and underweight in children aged between 6 and 59 months at the time of the interview. DHS data were collected by field workers after receiving intensive training lasting at least 4 weeks by the Ugandan Bureau of Statistics. The training included lectures, demonstrations of biomarker measurement or testing procedures, field practice with children at a health clinic, and standardization of height and weight measurements. Children of age 24 months and younger had their length measured in recumbent position but for ages above two years had their height measured while standing. The length was measured with the portable Harpenden Infantometer (range 30–110 cm, with digit counter readings precise to 1 mm), and height was measured using the Harpenden Portable Stadiometer (range 65–206 cm, digit counter reading precise to 1 mm). The z-scores for weight-for-age, weight-for-length/height, and length/height-for-age were provided in the DHS data calculated using the 2006 WHO Child Growth Standards [28]. A child was stunted, wasted, or underweight if he/she exhibited a z-score < -2 SD. Similarly, a child was defined as severely stunted, severely underweight, or severely wasted if the child exhibited a z-score < -3 [29].

### Main independent variables

Our primary variable of interest was VAD, defined as blood retinol-binding protein (RBP) < 17.325 ***μg***/mL, equivalent to 0.825 μmol/L. [30] The details of dried blood spot RBP measurements are described elsewhere [31]. In summary, blood collection for RBP testing was carried out only among children 6–59 months of age by collecting five blood spots from the finger/heel prick on a filter paper card. Blood samples were dried overnight, and stored at -20°C until tested. Testing was carried at the Department of Biochemistry at Makerere University in Kampala using retinol binding protein enzyme immunoassay method. Since RBP levels decrease during infection/inflammation, C-reactive protein (CRP), a standard measure of inflammation, was used to correct RBP values. CRP was measured using an ELISA commercial test kit (Bender MedSystems GmbH, Vienna, Austria) and a CRP ≤3 mg/L was considered to be normal. According to the WHO and CDC [32, 33], a serum CRP threshold of <5 mg/L is suggested to define normal values when using a rapid diagnostic test, or <3–10 mg/L when using immunoassays (e.g. ELISA). In the present study, an ELISA commercial test kit was used. The difference between the mean log RBP value for children with a normal CRP (≤3 mg/L) and the mean log RBP value for children with an elevated CRP (>3 mg/L) was back-transformed to provide an adjustment factor. RBP values for children with an elevated CRP were then multiplied by the adjustment factor to provide adjusted values. For children who were not tested for CRP, adjustment of RBP values for infection/inflammation was done using the method suggested by Thurnham et al. (2003). [34] The cutoffs values for RBP were derived using regression analysis methods of Engle-Stone and colleagues (2011) [35]. Using regression analysis methods, the cutoffs for VAD are <0.83 *μ*mol RBP/L (corresponding to <0.70 *μ*mol retinol/L) and is recommended by WHO [36].The sensitivity and specificity of derived cutoffs are 94.7 and 88.9% for children, respectively.

### Potential confounders

We used the WHO conceptual framework for the determinants of distal and proximal causes of childhood undernutrition to identify potential confounders of the relationship between VAD and undernutrition. [37] The proximal variables were defined at individual child and household-level, and the distal variables at the community or cluster-level. At the child level, vitamin A supplementation in the past 6 months, deworming medication in the past 6 months, diarrhea in the past 2 weeks, sex, and age of the child, anemia status, a combination of birth order and birth interval were included. Household variables were mother and father’s education and working status, wealth index quintiles, use of iodized salt, ownership of agricultural land, livestock, herd, or farm animals. Cluster/community level variables were the region-level place of residence (rural vs. urban), administrative geographical regions, growing season length in days, and the number of children under 5 years.

Anemia was determined using blood from a finger prick to measure hemoglobin concentration using an on-site battery-operated portable HemoCue analyzer. Hemoglobin concentration was categorized into anemia levels using WHO standards [38]: severe (hemoglobin concentration < 70 g/L), moderate (hemoglobin concentration between 70 and 99 g/L), or mild (hemoglobin concentration is between 100 g/L and below 110 g/L). Wealth index quintiles were calculated by the use of principal component analysis of household assets (household’s ownership of a number of consumer items such as television, car, radio, toilet facilities, flooring material and other characteristics related to wealth). The resulting asset scores were standardized in relation to a standard normal distribution with a mean of zero and a standard deviation of one. These standardized scores were used to create the breakpoints that define wealth quintiles as: lowest, second, middle, fourth, and highest. Specific information on the calculation of the wealth index quintiles including the syntax and the factor loadings is provided elsewhere. [39]

### Statistical analysis

We assumed intra-cluster correlation among the responses. We fitted generalized linear mixed-effects models assuming a binomial distribution with logit link function (logistic regression) while accounting for the primary sampling unit random effects (see equation in the supplemental documents) [40]. With the inclusion of the PSU random effects, we accounted for observable and unobservable factors specific to individuals within a primary sampling unit such as environmental factors (rainfall, temperature, and altitude), which potentially influence access to vitamin A-rich foods [41, 42]. Values are expressed as the mean ± standard deviation (SD), median and interquartile range in case of a skewed distribution, and as counts and percentages for categorical variables. Ninety-five percent confidence intervals (95% CI) for the prevalence were estimated using an exact binomial test. Between groups means or medians for continuous variables were compared using a 2-tailed 2-sample Student’s t-test or Wilcoxon rank-sum tests, respectively, and Pearson's chi-square or Fisher’s exact tests for comparisons of categorical variables. We tested the normality assumption for continuous variables with the Shapiro-Wilk test. [43].

**Fig 1:**
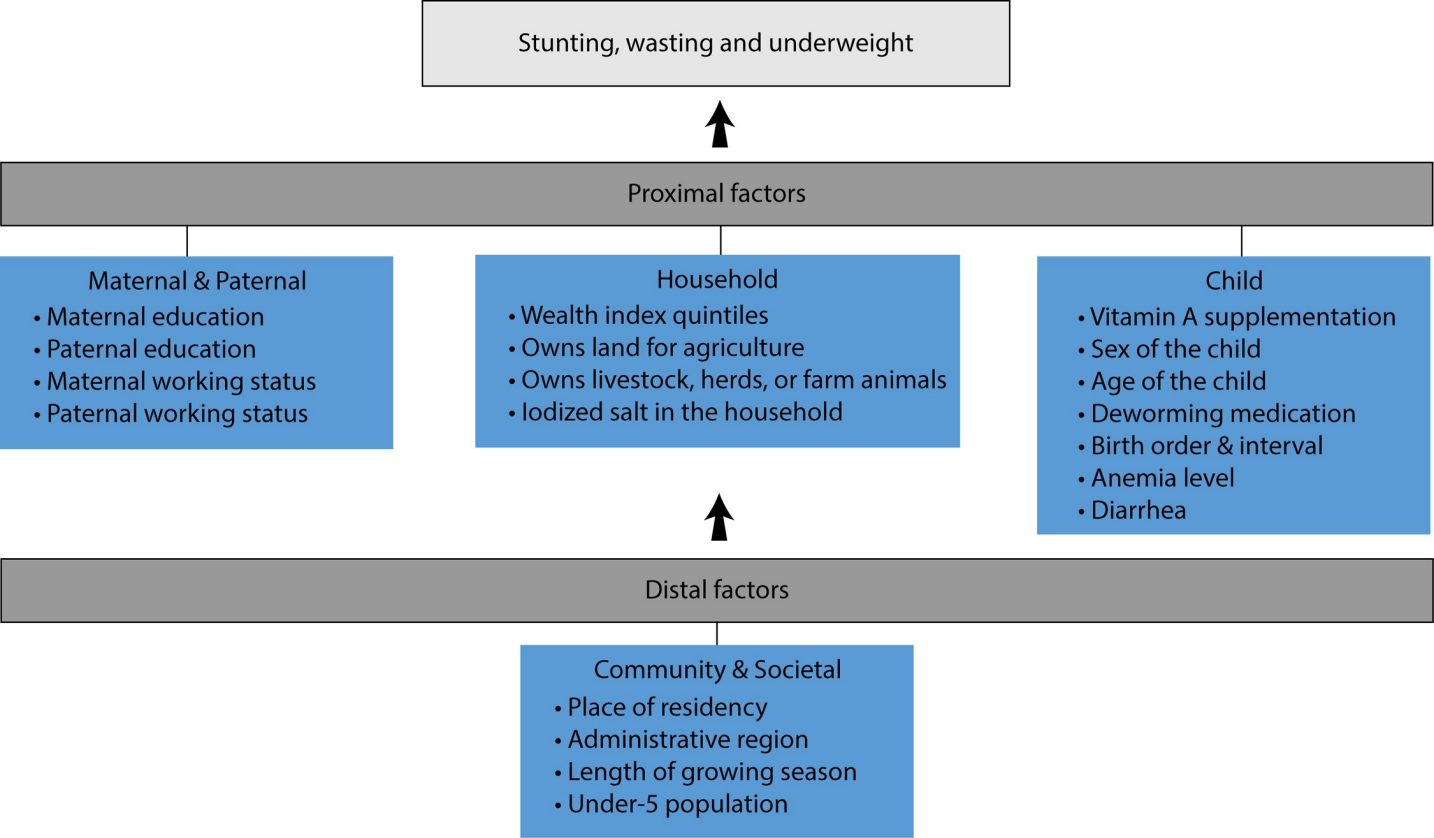
World health organization conceptual framework for the determinants of child undernutrition: Distal factors include community-level determinants, and proximal factors include child, household, maternal, and paternal-level determinants of undernutrition.

We reported the unadjusted and adjusted prevalence odds ratios (aOR) and 95% CI for VAD. We examined the association of vitamin A deficiency with binary outcomes of stunting, underweight, and wasting separately. In the multivariable-adjusted model, we adjusted for potential confounders identified from the WHO conceptual framework (**Fig 1**). Because the association of VAD with growth failure has been shown to be modified by age [23], we tested for potential interactions of VAD with age in the regression analysis.

To further minimize potential confounding bias, we performed a sensitivity analysis. First, we created propensity scores by modeling the odds of having VAD using the variables mentioned above. We then used the propensity scores as weights in the regression model [44]. Analyses were performed using SAS (version 9.4; SAS Institute Inc., Cary, NC) and R (version 3.0.1, R foundation for statistical computing, Vienna, Austria, *lme4 package*) [45]. A *p* < 0.05 was used to define statistical significance.

### Ethical considerations

The Uganda Demographic and Health Survey (UDHS) protocols and guidelines, including biomarker collection, were reviewed and approved by the Ugandan Ministry of Health (MOH) Ethical Review Committee and the Institutional Review Board of ICF International, USA. All research and methods were performed per the regulations and guidelines of the Ugandan MOH and ICF International, USA Ethical Review Committee, and the Institutional Review Board, respectively. Written informed consent was obtained from each participant, their parents or guardians before the survey. The DHS Program permitted the authors to use the data. The data are entirely anonymous; therefore, the authors did not seek further ethical clearance.

## Results

### Sample description

**Fig 2:**
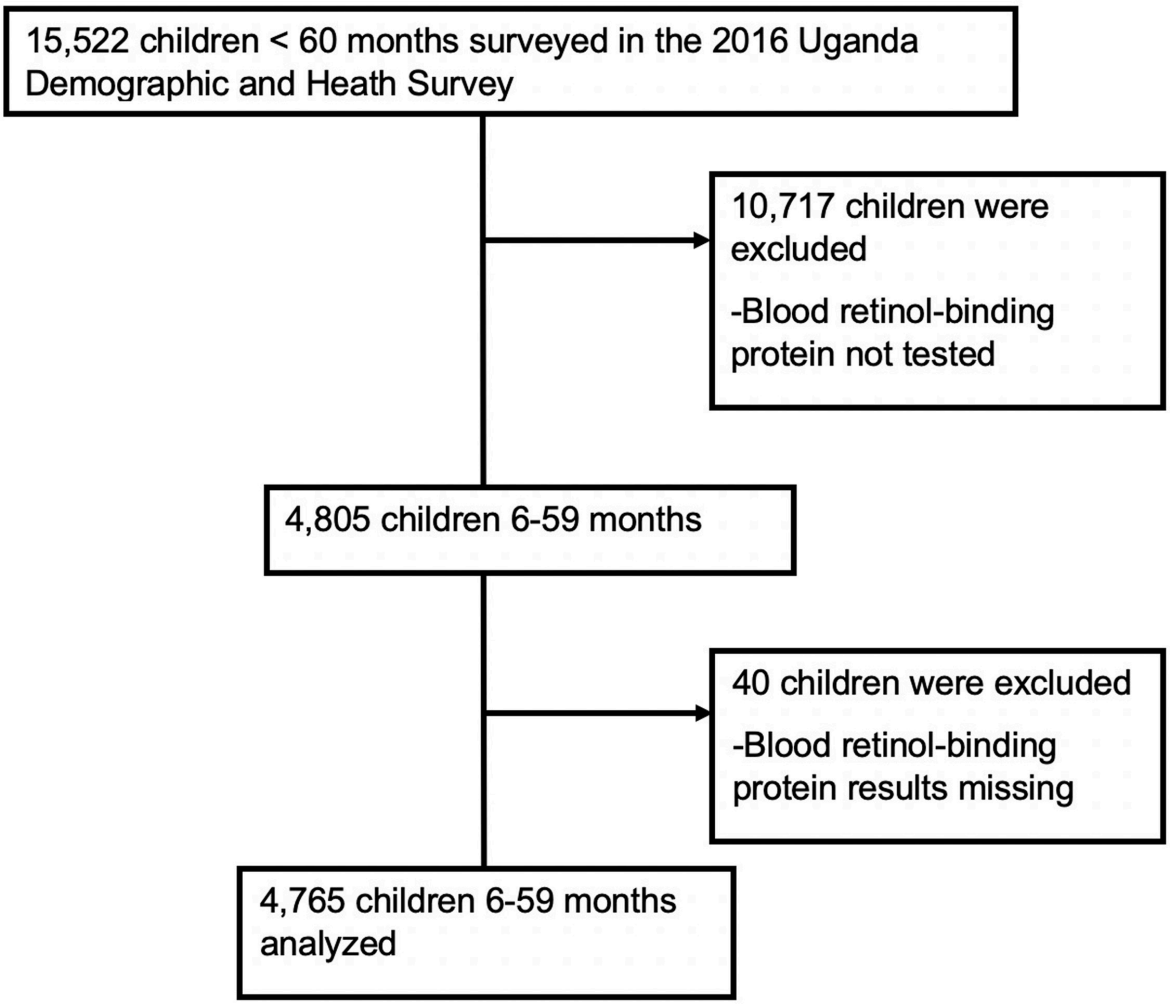
Flow of study participant selection.

**Fig 3:**
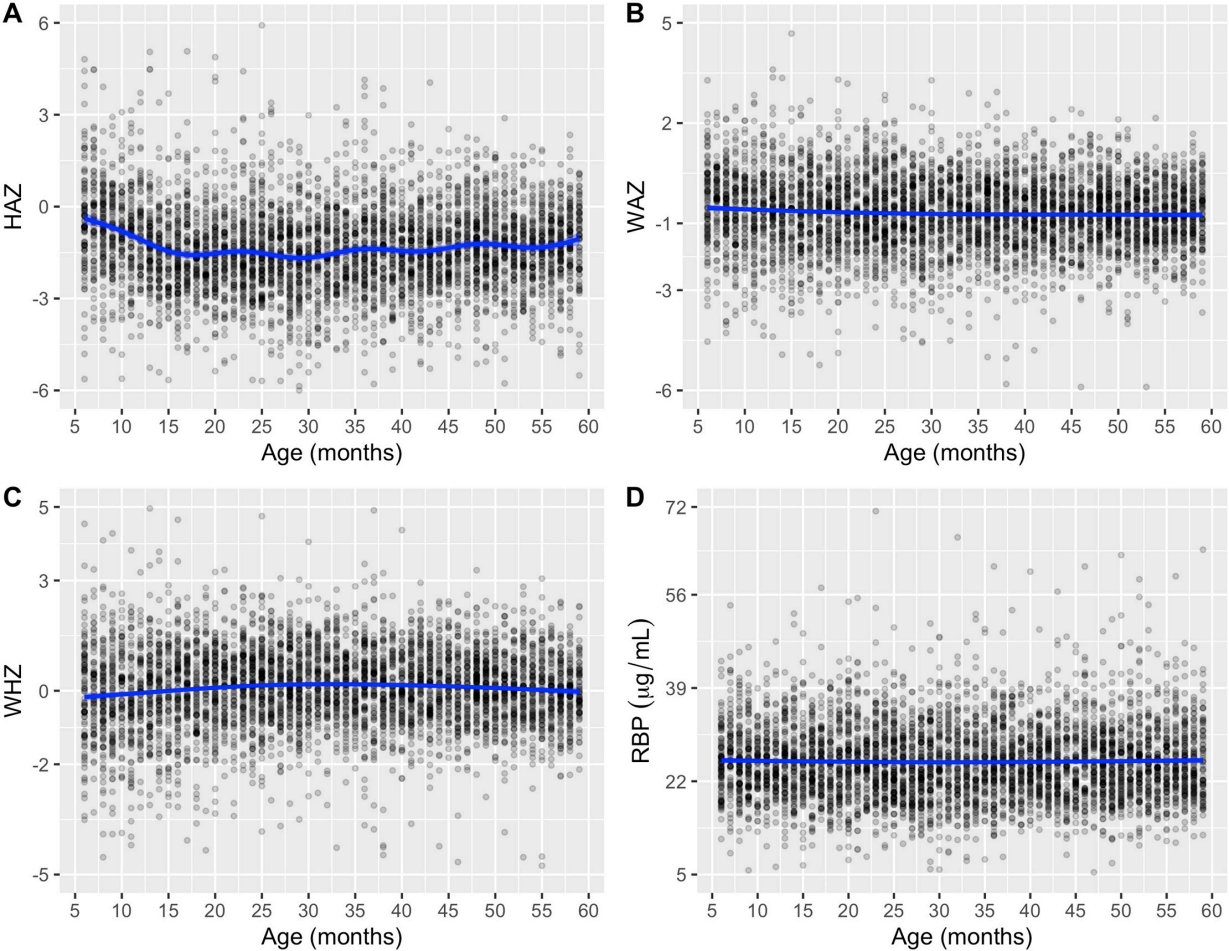
Z-score and blood RBP concentration as a function of age: Height-for-age Z-score (A), Weight-for-age Z-score, Weight-for-height Z-score (C) and serum RBP μg/mL in children aged 6–59 months. Data were fitted by thin plate regression spline. Growth faltering is seen according to the HAZ. Mean HAZ started below the standard with deficits ranging from -0.5 z-scores at 6 months to -1.5 z-scores at 18 months. Substantial variations were not observed in WAZ, WHZ, and RBP. Dots represent the individual child.

The flow of study sample selection is shown in **Fig 2.** The analyzed sample consisted of 4,765 children between the age of 6 months and 59 months. Data were collected between June 20, 2015, and December 16, 2016. The mean age was 32.5 months (SD = 15.5 months), and 50% were male (Table 1). Compared with children without VAD, higher proportions of children with VAD were of fourth or more birth order, birth interval less than two years, had moderate or severe anemia, had a mother who was not working, were from households with low wealth index and were from a rural residence. On a regional level, the proportions of children with VAD were highest in Busoga and Bukedia (17% each) and lowest in the Kigezi region (1.4%). No differences were seen in gender, mother's or father's education level, having received vitamin A supplementation (58% vs. 57%) or deworming medications in the previous 6 mo, having had diarrhea in the last two weeks, or coming from a household that owns the land for agriculture, owing livestock, herds or farm animals. Growth faltering is observed according to the HAZ (**Fig 3A**). Mean HAZ started below the standard with deficits ranging from -0.5 z-scores at 6 months to -1.5 z-scores at 18 months. Substantial variations were not observed in WAZ (**Fig 3B**), WHZ (**Fig 3C**), and RBP (**Fig 3D**).

**Table 1:** Background characteristics of the survey participants.

Characteristics	Overall N = 4765 (%)	Vitamin A Deficient n = 4341 (%)	Not Vitamin A Deficient n = 424 (%)	p-value
**Child-level variables**				
Child age, mo*	32.5±15.4	32.5±15.5	32.1±14.4	0.55
Categories of child age in mo				0.22
6–11	512 (10.7)	477 (11.0)	35 (8.3)	
12–23	1042 (21.8)	946 (21.8)	96 (22.6)	
24–59	3211 (67.5)	2918 (67.2)	293 (69.1)	
Male sex of child	2403 (50.4)	2173 (50.1)	230 (54.2)	0.10
Birth order and birth interval				0.03
First child	450 (31.8)	422 (32.6)	28 (23.9)	
2nd or 3rd child, > 2 years interval	590 (41.8)	529 (40.8)	61 (52.1)	
2nd or 3rd child, ≤ 2 years interval	99 (7.0)	92 (7.1)	7 (6.0)	
4th or more child, > 2 years interval	190 (13.5)	180 (13.9)	10 (8.6)	
4th or more child, ≤ 2 years interval	83 (5.9)	72 (5.6)	11 (9.4)	
Vitamin A supplementation in past 6 mo	1246 (59.4)	1142 (59.6)	104 (57.1)	0.65
Deworming medication in past 6 mo	2611 (61.2)	2382 (61.5)	229 (58.4)	0.17
Had diarrhea last two weeks	380 (18)	343 (17.9)	35 (19)	0.73
Anemia level				<0.01
None	2207 (46.4)	2049 (47.2)	158 (37.3)	
Mild	1142 (24.0)	1043 (24.1)	99 (23.3)	
Moderate	1298 (27.3)	1160 (26.8)	138 (32.6)	
Severe	111 (2.3)	82 (1.9)	29 (6.8)	
**Household-level variables**				
Wealth index quintiles				0.08
Lowest	1222 (25.6)	1114 (25.7)	108 (25.5)	
Second	1027 (21.6)	923 (21.3)	104 (24.5)	
Middle	960 (20.1)	871 (20.0)	89 (21.0)	
Fourth	879 (18.5)	798 (18.4)	81 (19.1)	
Highest	677 (14.2)	635 (14.6)	42 (9.9)	
Mother educated	3494 (86.4)	3200 (86.5)	294 (85.22)	0.43
Father educated	3121 (94.6)	2854 (94.7)	267 (93.4)	0.33
Mother working	3357 (75.2)	3084 (75.6)	273 (70.9)	0.04
Father working	3078 (93.3)	2816 (93.5)	262 (91.6)	0.23
Iodized salt in the household	4524 (94.9)	4122 (95.0)	402 (94.8)	0.27
Owns land for agriculture	3615 (75.9)	3298 (76.0)	317 (74.8)	0.58
Owns livestock, herds or farm animal	3452 (72.4)	3152 (72.61)	300 (70.75)	0.41
**Cluster-level variables**				
Place of residence				0.08
Urban	796 (16.7)	738 (17.0)	58 (13.7)	
Rural	3969 (83.3)	3603 (83.0)	366 (86.32)	
Administrative geographical region				<0.01
Kampala	165 (3.4)	154(3.6)	11 (2.6)	
Central 1	382 (8.0)	348(8.0)	34 (8.0)	
Central 2	390 (8.2)	353(8.1)	37 (8.7)	
Busoga	469 (9.8)	399(9.2)	70 (16.5)	
Bukedi	351 (7.4)	280(6.5)	71 (16.8)	
Bugishu	256 (5.4)	232 (5.3)	24 (5.7)	
Teso	342(7.2)	312 (7.2)	30 (7.1)	
Karamoja	232(4.9)	223 (5.1)	9 (2.1)	
Lango	333(7.0)	316 (7.3)	17 (4.0)	
Acholi	290(6.1)	266 (6.1)	24 (5.7)	
West Nile	299(6.3)	271 (6.2)	28 (6.6)	
Bunyoro	324(6.8)	300 (6.9)	24 (5.7)	
Tooro	414(8.7)	397 (9.2)	17 (4.0)	
Ankole	307(6.4)	285 (6.6)	22 (5.1)	
Kigezi	211(4.4)	205 (4.7)	6 (1.4)	
Growing season length, days†	315±15	300±15	302 ± 5	0.16
Under 5 population*	50 (26 to 91)	50 (25 to 92)	53 (30 to 88)	0.07

*n ± standard deviation;

†median (25^th^, 75^th^ percentiles)

The prevalence of VAD was 8.9% (95% CI: 8.1% to 9.6%, n = 424). Twenty-seven percent of children were stunted (95% CI: 26.1% to 28.6, n = 1302), 4% wasted (95% CI: 3.6% to 4.7%, n = 196), and 17% underweight (95% CI: 16.0% to 18.2%, n = 813). Stunting was more prevalent in children with VAD than in those without VAD (34.7% vs. 26.6%, p = 0.0004, **Table 2**). Similarly, severe stunting was more prevalent in the VAD population compared to those without VAD (15.7% vs. 9.4%, p = 005). Conversely, wasting and underweight were not associated with VAD (p <0.05).

**Table 2:** Prevalence of undernutrition of children 6–59 months by vitamin A deficiency status^1^.

		Vitamin A deficiency status		
Anthropometric indices	All Participants N = 4765	Yes N = 424	No N = 4341	p-value^2^
Stunting [no. (%)]	1302 (27.3)	147 (34.7)	1155 (26.6)	0.0004
Severe stunting [no. (%)]	467 (9.8)	58 (15.7)	409 (9.4)	0.005
Underweight [no. (%)]	813 (17.1)	84 (19.8)	729 (16.8)	0.11
Severe Underweight [no. (%)]	242 (5.1)	25 (5.9)	217 (5.0)	0.42
Wasting [no. (%)]	196 (4.1)	20 (4.7)	176 (4.1)	0.51
Severe Wasting [no. (%)]	74 (1.6)	7 (1.7)	67 (1.5)	0.86

The z-scores for height-for-age, weight-for-age, weight-for-length/height were calculated using the 2006 WHO Child Growth Standards. [28] A child was stunted, wasted, or underweight if he/she exhibited a z-score < -2 SD. Severe stunting, wasting, and underweight is z-score ≤ -3 SD.

^2^Fisher’s exact probability test for the association of vitamin A deficiency (yes/no) with each anthropometric indicator (yes/no) separately.

### Association between VAD and undernutrition indices

Children with VAD had 43% higher adjusted odds of stunted growth than those without (adjusted odds ratio, 1.43 (95% CI: 1.08 to 1.89, p = 0.01) (**Table 3**). Similarly, compared to those without, children with VAD had 64% higher odds of severe stunting (aOR 1.64, 95% CI: 1.14 to 2.35). No association was observed of VAD with wasting severe wasting, underweight, or severe underweight. A 1.5-fold increase in the stunting odds was consistently observed in the sensitivity analyses weighted with propensity scores (adjusted OR, 1.52 (1.16 to 1.99, p = 0.0005, **Table 4**). No significant interaction was observed between VAD and age.

**Table 3:** Associations between VAD and growth failure of children 6–59 months. Crude and multivariable-adjusted odds ratios and 95% confidence interval for the association of vitamin A deficiency with undernutrition.

Anthropometric indices	Unadjusted Odds Ratios of Undernutrition	Adjusted Odds Ratio of Undernutrition±
Stunting (N = 1302)	1.50 (1.19 to 1.89) *	1.43 (1.08 to 1.89) *
Severe stunting (N = 467)	1.58 (1.17 to 2.13) *	1.64 (1.14 to 2.35) *
Wasting (N = 196)	1.17 (0.73 to 1.89)	1.18 (0.69 to 2.04)
Severe wasting (N = 74)	1.03 (0.46 to 2.28)	0.84 (0.30 to 2.38)
Underweight (N = 813)	1.24 (0.95 to 1.62)	1.31 (0.95 to 1.80)
Severe underweight (N = 242)	1.22 (0.79 to 1.88)	1.23 (0.69 to 2.18)

*p<0.05

± Adjusted for vitamin A supplementation, deworming medication, diarrhea in the past two weeks, sex, and age of the child, anemia status, a combination of birth order and birth interval were examined in the study. Household variables included: mother and father’s education and working status, wealth index, use of iodized salt, ownership of agricultural land, livestock, herd or farm animals and community-level factors: level variables included the region-level place of residence (rural vs. urban), administrative geographical regions, growing season lengths in days, and under-five population.

**Table 4:** Sensitivity analysis for the association of VAD with undernutrition by VAD using propensity score weighted regression. Reported are crude and multivariable-adjusted odds ratios and 95% confidence interval.

Anthropometric indices	Propensity Score Adjusted Odds Ratio of Undernutrition
Stunting (N = 1302)	1.52 (1.16 to 1.99) *
Wasting (N = 196)	1.40 (0.78 to 2.50)
Underweight (N = 813)	1.34 (0.99 to 1.87)

*p<0.05

## Discussion

### Main findings

In this cross-sectional study of 4,765 children 6–59 months of age in Uganda, we found that children with vitamin A deficiency had higher odds of stunted growth than did those without. The association was independent of potential confounders at the child, household and village-level.

### Biological mechanisms

Several explanations have been proposed for the relationship between VAD and child growth failure. First, a causal relationship has been established between VAD and diarrheal diseases. Vitamin A is a critical micronutrient that maintains the structural and functional integrity of mucosal epithelial cells, including the gastrointestinal tract [46]. Disruption gastrointestinal mucosal integrity results in reduced nutrient absorption leading to growth failure and infectious diseases susceptibility. [15] Second, vitamin A controls cellular proliferation and differentiation through gene expression and thus has significant effects on immune cell proliferation and response. [47, 48] In 2013, the *Lancet* series on Maternal and Child Undernutrition estimated that over 2% of total deaths of younger than 5 years child is attributable to vitamin A deficiency, globally. [8] These deaths are primarily related to the increased susceptibility of children to diarrheal diseases and measles through dysregulation of the immune system during VAD. [49–51]. In a meta-analysis of 43 randomized clinical trials, oral vitamin A supplements in children aged 6 months to 5 years were associated with a 28% reduction in mortality attributable to diarrheal diseases. In this trial, vitamin A supplementation was also associated with a 15% reduction in the incidence of diarrheal diseases and a 50% reduction in the incidence of measles [7]. Lastly, retinoic acid, the active metabolite of vitamin A, is a gene regulator for growth hormone and is key in growth hormone secretion; therefore, the deficiency of vitamin A leads to the impaired synthesis and secretion of growth hormone in the pituitary growth hormone cells resulting in somatic growth failure, particularly in the preschool children. [52] It is possible that the VAD-stunting association observed in our study likely due to the direct effect of vitamin A on growth regulation or by increasing susceptibility to infectious disease.

### Comparisons with previous studies

Experimental animal studies demonstrate that vitamin A is essential for growth and maturation of epiphyseal-cartilage cells in long bones, and VAD induced cessation of epiphyseal growth in long bones. [53–55] Experimental and clinical studies show that ponderal growth is particularly affected only when the deficiency of vitamin A is very severe. [56, 57] The biological processes of the three nutritional status indicators in children: stunting, wasting, and underweight are somewhat different. Although stunting is a manifestation of chronic nutritional deficiency, wasting is instead a short-term (i.e., acute) response to inadequate intake or an infectious disease episode [58], and underweight is a combination of stunting and wasting. [59] We did not find an association of VAD with wasting and underweight. Furthermore, another study conducted in Uganda also found that the factors that were associated with stunting were different from those of underweight and wasting [60]. The lack of association between wasting and underweight with VAD could be explained by several factors. First, by the two types of selection bias: Length-biased sampling and Neyman bias (also known as incidence-prevalence bias, selective survival bias). [61] Regarding length-biased sampling, wasting is a severe acute form of malnutrition with poor prognosis among preschool children, resulting in higher mortality rates than stunting and underweight. [62] As a result, children with wasting are less likely to be selected in the study. In this cross-section study, prevalent cases rather than incident cases of undernutrition were sampled. On the other hand, stunting is a chronic form of malnutrition, and stunted children are more likely to be included in cross-sectional surveys. In our study, the prevalence of wasting was low (4%) compared to the prevalence of stunting (27%), thus limiting the statistical precision. Next, Neyman bias could have contributed to the null association we observed between wasting or underweight and VAD. For example, if VAD children with wasting or underweight die more frequently, the surviving cases will show a lower frequency of VAD, undervaluing the association of VAD with wasting and underweight. This kind of bias occurs only if the risk factor influences mortality from the disease being studied, such as VAD.

There are several advantages of using RBP as a surrogate marker for serum retinol: First, RBP is a protein, and therefore it can be detected with an immunologic assay, a simpler and less expensive method than high-performance liquid chromatography (HPLC) or spectrophotometry [63] analysis required to measure serum retinol. Second, RBP is more stable than retinol with respect to light and temperature, making it ideal for measuring in field settings and in resource-constrained settings. Third, RBP analysis requires a minimal amount of blood (10–20 μL), which is easily obtainable from a finger prick. On the other hand, venous blood is generally required to get enough volume for retinol analysis by HPLC. Of note, because of the high correlation between serum RBP concentration and serum retinol concentration (1:1 molar ratio), blood RBP concentration should reflect serum retinol concentration and, therefore, can be substituted for it as an indicator of vitamin A status.

Our results invite additional questions that are beyond the scope of the present study but might represent future directions for research. For instance, can linear growth failure, which is measured non-invasively in the field, be used as a surrogate marker for RBP or serum retinol to identify children in need of intense vitamin A supplementation or intervention? The logistical challenges of measuring serum retinol or RBP on population-level in LMICs such as Uganda can be daunting, and a non-invasive, inexpensive method to identify children in need of aggressive supplementation would be a helpful adjunct to nutritional interventions.

### Improving serum vitamin A status

Improvement of dietary intake of vitamin A and increased coverage of the vitamin A program are shown to improve vitamin A status of children in sub-Saharan Africa. It is well established that vitamin A supplementation is a highly cost-effective intervention for child health that provides a reliable source of vitamin A for preschool children in the face of economic instability, rising food prices, and inadequate dietary sources. High dose vitamin A supplementation programs have been established over the past three decades in many low- and- middle income countries to reduce preschool child morbidity, mortality, and blindness. Our finding showed the vitamin A supplementation program in Uganda to have a coverage rate of only 59% over the preceding 6 months, which below WHO recommended coverage rate of 85% [64]. Therefore, vitamin A supplementation programs that are tailored to high-risk regions and populations at risk of VAD are highly needed to reduce subclinical and clinical vitamin A deficiencies and to ensure adequate vitamin A status for preschool children.

### Strengths and limitations

There are several study strengths that must be acknowledged. First, we used data from a national survey of Uganda; hence the study findings are generalizable to the entire country. Second, the main study variables and the outcome were all measured by trained field workers, thereby reducing measurement bias. Third, RBP was adjusted for inflammation using CRP. The adjustment prevented overestimation of the prevalence of VAD in the presence of inflammation. Fourth, the selection of our variables was guided by the WHO contextual framework of undernutrition, and our analyses considered a variety of these sociodemographic and cluster-level characteristics that might potentially confound the relation between our primary study variable and the outcome. This adjustment also led to an improvement of the internal validity of the analyses of the primary study variable and attenuating potential confounding bias. Fifth, because the nutritional diversity, cultural and economic structure in Uganda correlates with most of sub-Saharan Africa, our findings have high external validity. They can be generalized to other populations in sub-Saharan Africa. Lastly, due to the strong evidence that infants <6 mo old have a lower serum RBP concentration than older infants and young children, [63]we limited our analysis to infants ≥6 mo of age, thereby mitigating misclassification error of VAD prevalence. Several limitations of this analysis should also be considered when evaluating the results. First, serum RBP-retinol relationship may be affected by various factors and conditions. These factors include systemic inflammation, protein-energy malnutrition, liver disease, and chronic renal failure. As thus, RBP may not 100% be saturated with retinol [65]. Second, the selection of the variables for the VAD-undernutrition association models was mainly driven by the availability of the information from the survey datasets. Therefore, other possible confounding variables that were not adjusted in our analysis could bias the observed association. Third, the cross-sectional nature of the study could not allow us to explore causation beyond associations.

## Conclusion

Our findings suggest a significant association of vitamin A deficiency with linear growth failure in preschool children in Uganda. Prospective studies to evaluate potential catch-up growth in vitamin A-deficient children following supplementation will further delineate the casual association and inform efforts to implement, monitor, and assess nationwide vitamin A supplementation in sub-Saharan Africa regions.

## Supporting information

S1 Appendix (DOCX)

## Abbreviations used

VAD: Vitamin A deficiency
RBP: Retinol binding protein
DHS: Demographic and health survey
HAZ: Height-for-age z-scores
WHZ: weight -for-height z-scores
WAZ: weight-for-age z-scores
