# Correction: Association of vitamin A deficiency with early childhood stunting in Uganda: A population-based cross-sectional study

**DOI:** 10.1371/journal.pone.0250138

**Published:** 2021-04-08

**Authors:** Paddy Ssentongo, Djibril M. Ba, Anna E. Ssentongo, Claudio Fronterre, Andrew Whalen, Yanxu Yang, Jessica E. Ericson, Vernon M. Chinchilli

In Table 1, the headings Vitamin A Deficient n = 4341 (%) and Not Vitamin A Deficient n = 4341 (%) are swapped in the third and fourth columns. The third column should be Not Vitamin A Deficient n = 4341 (%) and the fourth should be Vitamin A Deficient n = 424 (%). Please see the correct Table 1 here.

**Table 1:** Background characteristics of the survey participants.

Characteristics	Overall N = 4765 (%)	Not Vitamin A Deficient n = 4341 (%)	Vitamin A Deficient n = 424 (%)	p-value
**Child-level variables**				
Child age, mo*	32.5±15.4	32.5±15.5	32.1±14.4	0.55
Categories of child age in mo				0.22
6–11	512 (10.8)	477 (11.0)	35 (8.3)	
12–23	1042 (21.9)	946 (21.8)	96 (22.6)	
24–59	3211 (67.5)	2918 (67.3)	293 (69.1)	
Male sex of child	2403 (50.4)	2173 (50.1)	230 (54.2)	0.10
Birth order and birth interval				0.03
First child	450 (31.9)	422 (32.6)	28 (23.9)	
2nd or 3rd child, > 2 years interval	590 (41.8)	529 (40.9)	61 (52.1)	
2nd or 3rd child, ≤ 2 years interval	99 (7.0)	92 (7.1)	7 (6.0)	
4th or more child, > 2 years interval	190 (13.5)	180 (13.9)	10 (8.6)	
4th or more child, ≤ 2 years interval	83 (5.9)	72 (5.6)	11 (9.4)	
Vitamin A supplementation in past 6 mo	1246 (59.4)	1142 (59.6)	104 (57.1)	0.65
Deworming medication in past 6 mo	2611 (61.2)	2382 (61.5)	229 (58.4)	0.17
Had diarrhea last two weeks	380 (18)	343 (17.9)	35 (19)	0.73
Anemia level				<0.01
None	2207 (46.4)	2049 (47.3)	158 (37.3)	
Mild	1142 (24.0)	1043 (24.1)	99 (23.4)	
Moderate	1298 (27.3)	1160 (26.8)	138 (32.6)	
Severe	111 (2.3)	82 (1.9)	29 (6.8)	
**Household-level variables**				
Wealth index quintiles				0.08
Lowest	1222 (25.6)	1114 (25.7)	108 (25.5)	
Second	1027 (21.6)	923 (21.3)	104 (24.5)	
Middle	960 (20.2)	871 (20.1)	89 (21.0)	
Fourth	879 (18.5)	798 (18.4)	81 (19.1)	
Highest	677 (14.2)	635 (14.6)	42 (9.9)	
Mother Educated	3494 (86.4)	3200 (86.5)	294 (85.22)	0.43
Father Educated	3121 (94.6)	2854 (94.7)	267 (93.4)	0.33
Mother working	3357 (75.2)	3084 (75.6)	273 (70.9)	0.04
Father working	3078 (93.3)	2816 (93.5)	262 (91.6)	0.23
Iodized salt in the household	4524 (94.9)	4122 (95.0)	402 (94.8)	0.27
Owns land for agriculture	3615 (75.9)	3298 (76.0)	317 (74.8)	0.58
Owns livestock, herds or farm animal	3452 (72.4)	3152 (72.61)	300 (70.75)	0.41
**Cluster-level variables**				
Place of residence				0.08
Urban	796 (16.7)	738 (17.0)	58 (13.7)	
Rural	3969 (83.3)	3603 (83.0)	366 (86.32)	
Administrative geographical region				<0.01
Kampala	165 (3.5)	154(3.5)	11 (2.6)	
Central 1	382 (8.0)	348(8.0)	34 (8.0)	
Central 2	390 (8.2)	353(8.1)	37 (8.7)	
Busoga	469 (9.8)	399(9.2)	70 (16.5)	
Bukedi	351 (7.4)	280(6.5)	71 (16.8)	
Bugishu	256 (5.4)	232 (5.3)	24 (5.7)	
Teso	342(7.2)	312 (7.2)	30 (7.1)	
Karamoja	232(4.9)	223 (5.1)	9 (2.1)	
Lango	333(7.0)	316 (7.3)	17 (4.0)	
Acholi	290(6.1)	266 (6.1)	24 (5.7)	
West Nile	299(6.3)	271 (6.2)	28 (6.6)	
Bunyoro	324(6.8)	300 (6.9)	24 (5.7)	
Tooro	414(8.7)	397 (9.2)	17 (4.0)	
Ankole	307(6.4)	285 (6.6)	22 (5.1)	
Kigezi	211(4.4)	205 (4.7)	6 (1.4)	
Growing season length, days†	315±15	300±15	302 ± 5	0.16
Under 5 population*	50 (26 to 91)	50 (25 to 92)	53 (30 to 88)	0.07

*n ± standard deviation; †median (25th, 75th percentiles).
