# Supplementary material for: Association of vitamin A deficiency with early childhood stunting in Uganda: A population-based cross-sectional study
Source: PLoS One. 2020 May 29;15(5):e0233615. doi: 10.1371/journal.pone.0233615 (PMC7259702; doi:10.1371/journal.pone.0233615)
Supplement: S1 Appendix — (DOCX) [file pone.0233615.s001.docx]

**Supplementary online appendix**

**Appendix part A: Description of main dependent and independent measures**

**Outcome variables**

We focus on three child outcomes: stunting, underweight, and wasting at the time of the interview. All outcomes were defined as binary variables denoting whether a child’s *z*-score was less than –2: low height-for-age for stunting; weight-for-age for underweight or low weight-for-height for wasting. Separate models were estimated for stunting, underweight and wasting as dependent variables

**Independent variables**

The main independent variables were defined as binary variables denoting whether a child had vitamin A deficiency or not.

CRP-adjusted retinal binding proteins were used to calculate the binary value for vitamin A deficiency

**Appendix part B: Descriptive statistics**

This part of the appendix provides details for the variables analyzed in the article. Our choice of covariates was motivated by the WHO conceptual framework of causes of undernutrition. In the Table below are included distal and proximal variables associated with childhood undernutrition.

| **Variables** | **Categorization** |
| --- | --- |
| **Child-level variables** | |
| Age of the child | Categorized as 6 mo -<12 months, 12–<24 months, 24–59 months |
| Gender | Male, Female |
| Had diarrhea recently | Binary |
| Given deworming medication in past 6 mo | Binary |
| Combined birth order and birth interval | First child;  2nd or 3rd child, > 2 years interval;  2nd or 3rd child, ≤ 2 years interval;  4th or more child, > 2 years interval;  4th or more child, ≤ 2 years interval |
| Anemia | None, Mild, Moderate, Severe |
| Vitamin A supplementation in past 6 mo | Binary |
| **Household-level variables** | |
| Wealth index quintiles | Lowest, second, middle, fourth and highest |
| Iodized salt | Binary |
| Owns land for agriculture | Binary |
| Owns livestock, herds or farm animal | Binary |
| Maternal Education | Binary |
| Maternal working status | Binary |
| Father working | Binary |
| Paternal Education | Binary |
| Place of residence | Urban, Rural |
| **Cluster-level factors** | |
| Under 5 population | Continuous, median (25^th^ and 75^th^ percentile) |
| Growing season length | Number of days per year, mean, SD |
| Regions | Names of the major administrative regions of Uganda |

| **Distal/ cluster level Variable** | **Description** |
| --- | --- |
| Place of Residence | Rural vs urban |
| Under 5 population | The average number of people under the age of 5 (U5) within the 2 km (urban) or 10 km (rural) buffer surrounding the DHS survey cluster location. This is a high-resolution gridded population distribution estimates of the total number of people per pixel - broken down by male/female and 5-year age groupings. |
| Regions | Major administrative geographical regions of Uganda. |
| Growing season length | The number of days within the period of temperatures above 5°C when moisture conditions are considered adequate for crop growth. Under rain-fed conditions, the beginning of the growing period is linked to the start of the rainy season. The growing period for most crops continues beyond the rainy season, and, to a greater or lesser extent, crops mature on moisture stored in the soil profile. |

**The equation for GLMM model**

Suppose the outcome variable $Y_{ij}$ is the binary response for the $j^{th}$ participant within the $i^{th}$ cluster, where ${Pr(Y}_{ij}=1)$ is the probability that the $j^{th}$ participant, $j=1,2,\ldots,m_{i},$ within the $i^{th}$ cluster has growth failure and $1-{Pr(Y}_{ij}=1)$ is the probability that the $j^{th}$ participant within the $i^{th}$ cluster does not have growth failure. We assume a simple generalized linear mixed-effects model with one group:

$log\left\{ \frac{Pr\left[ Y_{ij=1} \right]}{1-Pr\left[ Y_{ij=1} \right]} \right\}=\mu+u_{i}$………………………………. (1)

where

$\mu$ is the overall mean; $u_{i}$ is the random effect for the $i^{th}$ cluster, $i=1,2,\ldots,C;$ the $u_{i}'s$ are $i.i.d \sim N\left( 0,\sigma_{B}^{2} \right)$.

$\sigma_{B}^{2}$ is the between-cluster (inter-cluster) variance. Another perspective of this model, in order to derive an expression for the intra-cluster correlation (ICC), is as follows. The random intercept logistic model for ${Y⃰}_{ij}$, a continuous variable, can be viewed as

${Y⃰}_{ij}=\mu+u_{i}+e_{ij}$………………………………………………………… (2)

where *Y_ij_* = 1 if ${\boldsymbol{Y}\boldsymbol{⃰}}_{ij}$>0 and 0 otherwise, and $e_{ij}$is assumed to have a logistic distribution with mean 0 and variance *π*^2^/3. Then the ICC is defined as the ratio of the between-cluster variance to the total variance, with the ICC given by $\frac{\sigma_{B}^{2}}{\sigma_{B}^{2}+\pi_{/3}^{2}}$.
